# The Reproductive Ecology of Industrial Societies, Part I: Why Measuring Fertility Matters

**DOI:** 10.1007/s12110-016-9269-4

**Published:** 2016-09-26

**Authors:** Gert Stulp, Rebecca Sear, Louise Barrett

**Affiliations:** 1 Department of Population Health, London School of Hygiene & Tropical Medicine, Keppel Street, London, WC1E 7HT UK; 2 Department of Sociology, University of Groningen / Inter-University Center for Social Science Theory and Methodology (ICS), Grote Rozenstraat 31, 9712 TG Groningen, The Netherlands; 3 Department of Psychology, University of Lethbridge, Lethbridge, AB T1K 3M4 Canada

**Keywords:** Fertility, (mal)adaptive behavior, Industrial population, Secondary database, Researcher degrees of freedom, Family

## Abstract

Is fertility relevant to evolutionary analyses conducted in modern industrial societies? This question has been the subject of a highly contentious debate, beginning in the late 1980s and continuing to this day. Researchers in both evolutionary and social sciences have argued that the measurement of fitness-related traits (e.g., fertility) offers little insight into evolutionary processes, on the grounds that modern industrial environments differ so greatly from those of our ancestral past that our behavior can no longer be expected to be adaptive. In contrast, we argue that fertility measurements in industrial society are essential for a complete evolutionary analysis: in particular, such data can provide evidence for any putative adaptive mismatch between ancestral environments and those of the present day, and they can provide insight into the selection pressures currently operating on contemporary populations. Having made this positive case, we then go on to discuss some challenges of fertility-related analyses among industrialized populations, particularly those that involve large-scale databases. These include “researcher degrees of freedom” (i.e., the choices made about which variables to analyze and how) and the different biases that may exist in such data. Despite these concerns, large datasets from multiple populations represent an excellent opportunity to test evolutionary hypotheses in great detail, enriching the evolutionary understanding of human behavior.

Today’s industrialized world, with its selfie-sticks, cat cafés, and pilotless planes, differs quite remarkably from the world we occupied only a few decades ago. The contrast is, of course, even more stark when we reflect on the environments we occupied a few hundred thousand years ago, as hunter-gatherers on the African plains. From an evolutionary perspective, a selfie-stick is, arguably, a trivial addition to modern life, but other changes undoubtedly have influenced evolutionary processes: we have vastly reduced rates of child mortality, substantially increased our lifespans, and many countries now display below-replacement levels of fertility.^1^

Given this state of affairs, it is not surprising that humans are often considered to have transcended their biological heritage, resulting in the view that evolutionary processes are irrelevant to an understanding of human behavior. Many social scientists who study fertility rarely, if ever, view their work through an evolutionary lens (see van den Berghe 1990; Cochran and Harpending 2009; Kaplan 1996; Morgan and King 2001; Sear 2015a; Turke 1989; Udry 1996; Wilson 1999). Whether an evolutionary approach is essential to the study of fertility is not our focus here (for a review, see Sear 2015b). Rather, we address the claim that measures of fitness in contemporary populations (or their proxies, such as fertility) cannot offer any insight into evolutionary processes. In particular, many evolutionary psychologists argue that an evolutionary approach should concentrate on the outcomes of past selection, identifying the adaptations that suited us to a hunter-gatherer niche (e.g., Barkow et al. 1992; Cosmides and Tooby 2013; Symons 1990; Tooby and Cosmides 2005). Not all evolutionarily minded researchers agree, of course, and there is long-standing debate on this issue: during the late 1980s and early 1990s in particular, the value, or lack thereof, of studying fertility differentials was fiercely contested, with human behavioral ecologists on one side and evolutionary psychologists on the other (Alexander 1990; Barkow 1990; Betzig 1989; Caro and Borgerhoff Mulder 1987; Crawford 1993; Smuts 1991; Symons 1989, 1990; Tooby and Cosmides 1990; Turke 1990a, b; see also Smith et al. 2001).

Here, we revisit this debate, arguing that the measurement of fertility in contemporary populations is integral, and not irrelevant, to evolutionary psychology. This is because the study of fertility can provide empirical evidence to support the (usually untested) assumption of adaptive mismatch that is central to much evolutionary psychological theorizing (Barkow et al. 1992; Confer et al. 2010; Deaner and Winegard 2013; Geher 2013; Tooby and Cosmides 2015), by highlighting how and why people fail to maximize fitness. In other words, the study of present-day fertility can and does enrich our understanding of evolutionary processes, despite the fact that, or even precisely because, fertility behavior in industrial societies may be maladaptive. We also emphasize that understanding fertility among industrial societies will enrich the study of human behavioral ecology as well: it is clear that the blanket term “industrial society” masks a considerable amount of variation, thus offering the opportunity to conduct comparative research that complements the array of studies conducted on small-scale societies. We should not, therefore, treat industrial society as a monolith, but recognize that industrial settings consist of a number of distinct “ecologies” that offer a rich source of insight into how and why reproductive decision-making varies with environmental circumstances. In the second half of our paper, we discuss some of the benefits and issues associated with analyzing large-scale databases that are commonly used to study fertility—concerns that may not be recognized by those not well acquainted with these kinds of data sources. The companion paper (Stulp et al. 2016, this issue) provides an empirical example of how to handle the challenges of using large databases, via an analysis of the relationship between wealth and fertility in the contemporary United States.

In what follows, we define *reproductive ecology* as the evolutionary study of reproductive strategies and decision-making that is responsive to ecological context (in line with Ellison 1994; Jasienska 2013; Voland 1998). Life history trade-offs and (adaptive) physiological mechanisms are considered essential to an understanding of how reproduction is regulated (Voland 1998), but we do not consider physiological mechanisms explicitly here (see Ellison 1994). In addition, although our focus is exclusively on fertility among industrialized populations, many of our points hold equally well (sometimes even more so) for nonindustrial populations, ranging from small-scale societies to “transitioning” populations (e.g., Alvergne et al. 2013; Bolund et al. 2015; Colleran et al. 2014; Gibson and Lawson 2014; Moorad 2013; Ross et al. 2016; Shenk et al. 2013). A number of the other articles in this special issue also attest to this. Industrial societies, however, are considered particularly peculiar from an evolutionary perspective, so we begin by outlining the reasons why it is important to characterize fertility profiles and reproductive differentials in such populations, before going on to discuss the specific challenges of studying fertility in industrial settings.

## On Why We Should Study Fertility within Industrial Societies

### Because Natural Selection Continues to Operate

One frequent claim made for why natural selection is no longer relevant to industrial society is because of advances in modern medicine, and a consequent reduction in mortality rates (see discussions in Bolund et al. 2015; Stearns et al. 2010; Tait 1869; Zampieri 2009). These ideas have entered popular culture and influence how the subject is presented to the public at large: the renowned naturalist and television presenter Sir David Attenborough said recently, “We stopped natural selection as soon as we started being able to rear 90–95% of our babies that are born. We are the only species to have put a halt to natural selection” (Meikle 2013). Assertions such as this rest on the assumption that drastically reduced variation in health and mortality renders natural selection no longer “effective”—in other words, it fails to eliminate those individuals who would not have been expected to survive and reproduce in earlier eras. Similar but weaker claims about the power of natural selection are made by adherents of the “Santa Barbara” school of evolutionary psychology (e.g., Barkow et al. 1992; Tooby and Cosmides 2015), and it is this stance in particular that we counter here. Specifically, this evolutionary psychological theorizing suggests that natural selection has reduced in importance since the dawn of agriculture because subsequent rates of cultural change have been too rapid for genetic evolution to keep pace (with the technologies that lead to reduced mortality being a prime example). Although both of these claims may contain a kernel of truth, the point here is that they should be tested empirically, not taken as axiomatic.

It is also vital to remember that natural selection acts on differential reproduction, and that differential survival is just one of the forces that reduces opportunities to propagate genes. That is, although low rates of (child) mortality may result in reduced variation for this component of fitness, variation in fitness because of fertility may still be significant. Importantly, it is *relative fitness* that matters most to evolutionary processes (i.e., an individual’s fitness scaled to the fitness of the rest of the population: e.g., Orr 2009). Put differently, it is variation in (relative) fitness that is relevant to an assessment of natural selection, rather than levels of mortality in a population or the mean number of surviving children per individual. Moreover, it is important to realize that, in order for a genetic response to occur, the fitness-related trait of interest must be (genetically) inherited, and crucially, there must be a genetic association between the trait and relative fitness (Bolund et al. 2015; Mills and Tropf 2015; Morrissey et al. 2010; Orr 2009; Tropf et al. 2015b). When heritability (referring to the amount of phenotypic variation in a trait that can be explained by genetic variation) is high, the genetic response to natural selection on a trait is likely to be stronger than when heritability is low. Thus, far from natural selection being weakened in industrial settings, it may, in fact, act more strongly on particular traits (see also Bolund et al. 2015; Tropf et al. 2015a, b; Udry 1996). This is because the heritabilities of certain traits are likely to be much higher now than in the past since the environment has become more homogenous (owing to universal health care, vaccination programs, etc.; see Bolund et al. 2015; Bras et al. 2013; Kohler et al. 2002; Tropf et al. 2015a; Udry 1996).

Indeed, a number of researchers have argued that natural selection has been stronger since the advent of agriculture, precisely because of large cultural shifts (Cochran and Harpending 2009; Hawks et al. 2007), and there is strong evidence for selective sweeps (Hawks et al. 2007; Mathieson et al. 2015; Pritchard et al. 2010; Turchin et al. 2012; see also Bolhuis et al. 2011; Laland and Brown 2011). Others have taken up the challenge of assessing the strength of natural selection in contemporary populations and, using phenotypic, genetic, or pedigree data, have found evidence for selection on various traits, including age at first birth and height (e.g., Byars et al. 2010; Stearns et al. 2010; Stulp et al. 2012a, b, c, 2015; Tropf et al. 2015b). Note that although such studies are highly suggestive, they are not conclusive since, typically, lifetime reproductive success is used. Such proxies do not reflect fitness in any direct sense because (*a*) this is not a measure on the molecular genetic level, and thus changes in allele frequencies cannot be established; (*b*) lifetime reproductive success may be a poor proxy for a more long-term measure of fitness because of life history trade-offs: high levels of fertility in the current generation may come at a cost to future fitness (Stearns 1992; see Lynch 2016 for a recent example in an industrialized population); and (*c*) using lifetime reproductive success as a measure of fitness is inappropriate when the population is not stationary but either growing or shrinking, or when population growth is density-dependent (e.g., Baldini 2015; Jones and Bird 2014; Jones 2015; Low et al. 2002). For example, a recent analysis has shown that a well-timed birth in a growing population may have a greater influence on fitness than the production of additional numbers of children (Jones and Bird 2014).

These caveats aside, it is clear that empirical assessments are needed to determine whether natural selection is operating in contemporary populations or whether the strength of, or response to, such selection has changed over time (see also Scranton et al. 2016); measuring fitness-related traits such as fertility and lifetime reproductive success is an obvious place to start such an investigation. Thus, the idea that natural selection has been of limited importance in more recent times seems to have little empirical support given the highly suggestive nature of the work cited above. On-going developments in genomics will be able to contribute to these topics in the not too distant future (see also Chen et al. 2016; Fieder and Huber 2016), allowing us to look at past selection by examining a large set of current genomes, or by comparing genomes sampled historically across generations.

### Because People Continue to Make Reproductive Decisions (Even If They Don’t Maximize Fitness)

In the debates of the 1980s and 1990s, evolutionary psychologists argued that organisms do not, and could not, possess any kind of generalized fitness-maximizing mechanism, and therefore, they considered the contemporary study of fitness outcomes irrelevant to the study of evolutionary adaptation (Barkow et al. 1992; Cosmides and Tooby 1997; Symons 1989, 1990; Tooby and Cosmides 1990). Instead, these evolutionary psychologists argued that evolutionary analyses should focus on the psychological mechanisms that underpin behavior, along with the environmental cues to which they are responsive. This in turn raises the possibility that environmental change, if it occurs rapidly and is drastic enough, will result in maladaptive behavior. Indeed, evolutionary psychologists argue that differences between human psychological mechanisms and the cues available in the post-Pleistocene industrial environments are sufficiently large to generate a mismatch between our evolved psychology and our behavior. Consequently, it is deemed unlikely that we continue to act in fitness-enhancing ways. This is why the study of fitness differentials in contemporary populations (“counting babies”; Crawford 1993, 2000) is deemed uninformative: such studies can neither identify underlying mechanisms nor are they likely to identify the optimal fitness-enhancing strategy (since such strategies can no longer result in adaptive behavior). Although the outright dismissal of the “counting babies” approach is much less common today, the idea that modern human behavior is necessarily mismatched to the environment is a regular feature of much work in evolutionary psychology (e.g., Confer et al. 2010; Deaner and Winegard 2013; Geher 2013; Tooby and Cosmides 2015; Van Vugt et al. 2008), and consequently, people’s fertility behavior is rarely measured. There are also more recent (and on-going) discussions about whether psychometric measures are more informative than biometric measures, which tend to mirror these earlier debates (e.g., Copping et al. 2014; Dunkel et al. 2015; Figueredo et al. 2015). Claims therefore continue to be made that measuring fertility should be foregone in favor of studying other, allegedly more informative traits (for instance: “In modern times, mating success must be used as a proxy for [reproductive success]” [Camargo et al. 2013:138], a statement that contains the implicit, and wholly untested, assumption that current mating success is a better proxy for ancestral fitness than current fitness).

Counters to these evolutionary psychological arguments have been made repeatedly (Alexander 1990; Betzig 1989; Caro and Borgerhoff Mulder 1987; Irons 1990; Smuts 1991; Turke 1990a, b). Turke (1990a, b), for example, argues convincingly that documenting the environments in which adaptive behavior does or does not occur provides a powerful means of identifying the potential cues or mechanisms that result in (non-)fitness maximizing behaviors. Such data can therefore provide the empirical grounding for evolutionary psychologists’ claims that shifts in the nature of environmental cues can and will result in maladaptive behavior. After all, if we do not collect measures of fitness, the idea that behavior in industrial settings is maladaptive is simply an unsupported assumption (see also Stulp and Barrett 2016b). Moreover, investigations into psychological mechanisms alone are insufficient (Alexander 1990): if novel cues feed into psychological mechanisms but have no adverse effects on fitness outcomes, then, by definition, the behavior is not maladaptive. Strong claims regarding maladaptation and mismatch should thus reflect the findings of relevant behavioral measures and fitness outcomes and should not be based on mere speculation or simply by highlighting differences between the supposed ancestral world and contemporary environments (see also Smith et al. 2001; Zuk 2013). Studying fertility behavior in contemporary populations is therefore worthwhile because it allows us to get a better understanding of the environmental cues that feed into reproductive decision-making, regardless of whether that decision-making turns out to be adaptive or maladaptive in the long run.

In contrast to the evolutionary psychological view, human behavioral ecologists are interested in behavioral strategies and their functional outcomes—in other words, the extent to which they contribute to survival and reproductive success. As such, their approach requires the measurement of fitness-related traits, including fertility. Although many human behavioral ecologists recognize the potential for an evolutionary mismatch, central to the behavioral ecological approach is the application of the *phenotypic gambit* and, more recently, the *behavioral gambit* (Borgerhoff Mulder and Schacht 2012; Fawcett et al. 2013; Grafen 1984; Rittschof and Robinson 2014): the assumption that there are no (or very few) constraints (genetic or otherwise) on humans’ ability to arrive at a fitness-maximizing solution. That is, humans are argued to be sufficiently plastic to track environmental changes in fitness-enhancing ways (Borgerhoff Mulder and Schacht 2012), especially given that we have engineered those environments for ourselves via a process of niche construction (Laland and Brown 2006). Flexible learning mechanisms allow individuals to identify benefits, costs, and trade-offs in a given environment and, in doing so, to behave adaptively and maximize their fitness. Thus, behavioral ecologists consider the adaptiveness of behavior in post-Pleistocene and industrial environments to be an empirical issue (e.g., Borgerhoff Mulder 1998). Of course, there may be limits to humans’ ability to respond adaptively to change, but these limits cannot be predicted a priori.

Indeed, it seems fair to say that behavioral ecologists have assumed that the behavior of humans in industrialized populations is unlikely to be fitness-maximizing, based on low fertility and high rates of childlessness. This, in turn, may partly explain why such populations have been under-studied from a behavioral ecological perspective, relative to small-scale societies (Sear et al. 2007). Indeed, many early studies that aimed to explain low fertility were theoretical accounts examining whether this could be adaptive in the long-run (e.g., Boone and Kessler 1999; Hill and Reeve 2005; Mace 1998; Rogers 1990), rather than empirical studies of people’s actual behavior. Having said this, there has been a noticeable upturn in the number of empirical studies conducted in industrial populations over the past decade or so (Nettle et al. 2013). This may be ascribed to several developments: (*a*) major increases in computing power, which has made sophisticated statistical modelling of the kind needed for such analyses more tractable and within the reach of almost all researchers; (*b*) the increased availability of longitudinal databases that contain sufficient data to allow for evolutionarily based analyses: many now contain a sufficient sample of individuals who have completed reproduction, and some are even multigenerational; (*c*) the expansion of human behavioral ecologists into disciplines beyond anthropology, which formed their original home, and where the study of cultural diversity naturally included a greater focus on small-scale societies; and (*d*) increased awareness that such large datasets from industrial populations provide an excellent means for testing evolutionary ideas. Goodman et al. (2012), for example, used a unique multigenerational dataset from Sweden to test whether limiting fertility was an adaptive strategy (i.e., increased the number of [great-] grandchildren). Their results suggest that fertility limitation resulted in reduced fitness over the long term and hence was maladaptive, although there was also evidence to suggest that the descendants of larger families suffered present-day costs in terms of lower social status and educational achievement.

It may seem odd to suggest, on the one hand, that measuring present-day fertility enriches our understanding of mechanisms underlying (mal) adaptive behavior and, on the other, that establishing whether behavior is adaptive can be achieved only via long-term, multigenerational studies. This apparent contradiction is resolved once we appreciate that these points deal with different levels of explanation. As natural selection is an outcome rather than a process (Endler 1986), it is only possible to assess its effects on a given trait retrospectively. The processes and decisions that lead to particular outcomes, on the other hand, are based on cues received from the current environment. Although humans use foresight and planning, they do not use these abilities to behave in ways that precisely map onto the long-term outcome of selection (i.e., people are not looking five generations into the future and making their decisions accordingly, nor are they able to). In this, then, we agree with the evolutionary psychologists’ position that organisms do not possess any form of general-purpose “fitness maximizing” mechanism (e.g., Tooby and Cosmides 1990)—and, to be fair, this is also the position of most other behavioral ecologists. Instead, selection produces organisms possessed of mechanisms that are sensitive to particular kinds of environmental information, and it is the operation of such mechanisms that results in fitness-enhancing behavior (ranging from the rather fixed abilities to see or hear certain frequencies to highly flexible mechanisms, including individual and social learning and planning abilities). Thus, studying fertility behavior, and establishing how this is influenced environmentally (in terms of both conscious decision-making as well as other physiological and unconscious psychological mechanisms), will lead to a better understanding of the mechanisms involved. The question of whether such decisions are adaptive in the long run is therefore a related but separate evolutionary question.

The desire to gain a better understanding of fertility behavior and its drivers, rather than just measures of fitness, increases the scope of our investigations: not only can we study the number of (surviving) children born to an individual, we can also examine birth intervals, parity-specific progression (e.g., the likelihood of becoming a parent, or having a second or third child), within-couple fertility, and multi-partnered fertility, because different mechanisms likely feed into these different behaviors (Billari et al. 2009; Namboodiri 1972). Fertility norms, for instance, may explain why many couples end up having two children in contemporary populations, whereas individual circumstances (e.g., health, wealth) and previous experiences may affect birth intervals or parity-specific progression (see Stulp and Barrett 2016b for further discussion). These measures also provide a basis for comparison with small-scale societies. For instance, whereas the length of the interbirth interval may be a good indicator of overall reproductive output in high-fertility populations (where intervals are heavily influenced by the nutritional status and health of the mother), this may not be true for industrialized populations where nutritional concerns are less important. In the UK, for instance, an important determinant of interbirth interval and fertility is the age at first birth: highly educated women in the UK have later first births and fewer children than less-educated women but tend to progress more rapidly to subsequent births (Berrington et al. 2015; Rendall and Smallwood 2003).

### Because It Connects an Evolutionary Approach to the Broader Social Sciences

With their increasing focus on industrial populations, behavioral ecologists are catching up with demographers, economists, and sociologists who have been attempting to understand patterns of fertility (and particularly, patterns of fertility decline) over the past two centuries (e.g., Balbo et al. 2013; Becker 1960; Lee 2003; Sear et al. 2016). Work in all these fields makes it apparent that cultural evolutionary processes need to be considered as part of any kind of evolutionary approach when examining temporal patterns of fertility variation. There is a general consensus among demographers (Bongaarts and Watkins 1996; Bras 2014; National Research Council 2001; Pollak and Watkins 1993), for example, that explanations for low fertility should be sought in a combination of economic reasons (including considerations of lower child mortality and increased costs of rearing children) along with the diffusion of novel ideas through social interactions (e.g., use of contraception)—ideas that are also heavily associated with work in behavioral ecology and cultural evolution.

Evidence that the decline in fertility cannot be explained solely by economic factors is argued to lie in the specific cultural and geographical patterns of fertility decline: fertility behavior is more similar in geographically connected areas (Goldstein and Klüsener 2014), and in areas that are linguistically similar (van Bavel 2004), regardless of economic circumstances. It has also been found that culturally distinct groups, such as religious groups (McQuillan 2004), possess characteristic pro- and anti-natal attitudes that can persist over time and temper the influence of economic factors. There is also more direct evidence of such social influences on fertility: Colleran et al. (2014), for instance, have shown that the characteristics of people in a woman’s social network can exert a stronger influence on her fertility behavior than her own characteristics (see also Colleran 2016). Similarly, Balbo and Barban (2014) have shown that the fertility decisions of individuals are influenced by the decisions of their friends. Structural changes in society, changing social influences, and the spread of novel ideas through networks, may explain some of the quirks in contemporary industrialized populations, such as an increasing number of individuals who have actively (and happily) decided to be child-free throughout their entire lives, despite having the economic means to raise children (Nazarinia Roy et al. 2014)—something that is difficult to reconcile with classic behavioral ecological principles (see also Stulp and Barrett 2016b). The increasing number of ethnographic and mixed-method accounts of reproductive decision-making in industrial societies (e.g., Bernardi et al. 2014; Cooper 2014; Edin and Kefalas 2005) can provide the kind of qualitative detail needed to flesh out quantitative patterns derived from large-scale surveys. Fertility behavior, then, is a topic that can provide a bridge from evolutionary approaches to the broader social sciences, with each field offering a unique perspective on a behavior that cannot be fully understood within a single framework.

Most important in this respect, the large body of literature on fertility in demography and sociology makes it abundantly clear that the industrial world is not a monolith. Across industrial populations there is wide variety in cultural background as well as the nature of social institutions, both of which markedly affect behavior. For instance, the extent to which female earnings are associated with the probability of becoming a parent or having further children depends on population-specific policies, such as the availability of childcare, the length of maternity (and paternity) leave, and whether or not it is paid leave (see Stulp and Barrett 2016b for review). We want to make clear, therefore, that although this paper makes broad points about “industrial societies,” we do not consider such societies to form a single homogeneous unit. This can be seen as an application to fertility research of Henrich and colleagues’ warning about the overreliance on WEIRD (Western, Educated, Industrialized, Rich, and Democratic) populations in psychology (Henrich et al. 2010). In addition to avoiding the assumption that WEIRD populations can be taken as representative of human populations in general, we should also avoid the assumption that any single WEIRD population can be taken as representative of all industrialized, economically prosperous societies as a whole. For example, a recent meta-analysis concluded that there was a significant association between father absence in childhood and age at menarche (Webster et al. 2014), a topic of current interest in evolutionary circles. However, not only was every study included in the meta-analysis conducted on a WEIRD population, but the majority were also English-speaking (22 of 29 populations where the location of the study could be identified). The idea of the industrial world as representing a single entity was also very apparent in this review, as some of the research cited did not even specify the study population used (which may also reflect the assumption that human populations throughout evolutionary and historical time have remained essentially unchanged, and will mount a single, universal response to similar circumstances). Just as evolutionary generalizations based only on WEIRD data from the industrial world are no longer excusable given the well-documented diversity in cognitive processes and behavior (Henrich et al. 2010), generalizations across “the industrial world,” and even across different social strata within a population, are similarly on shaky ground (suggestions also made by Henrich and colleagues).

Measuring fertility within, and acknowledging variability between, industrial populations is also relevant to comparative work, allowing for rigorous cross-population and cross-species comparisons that further our understanding of life history evolution. Such comparisons will obviously be incomplete if they do not incorporate the full range of human lifeways, which means industrial settings must be considered. Indeed, this may reveal patterns that are missed when industrial populations are excluded. For example, analyses by Burger et al. (2011), Burnside et al. (2012), and Moses and Brown (2003) show that fertility behavior within and between industrialized populations is in line with macro-ecological patterns observed across nonindustrialized human populations and other mammalian species, suggesting that industrialized societies are not an evolutionary anomaly.

Fertility behavior is thus a key area in which to study the intersection of human behavioral ecology and evolutionary psychology with other evolutionary research areas, such as comparative life history and cultural evolution. As noted above, evolutionary approaches can also add value to the studies performed by demographers, economists, and sociologists, which can help to further contextualize evolutionary theorizing. The study of fertility thus encompasses all areas of evolutionary research, as well as the different disciplines within the social sciences, allowing for a fully biocultural understanding of a key human behavior.

## The Use of Secondary Data in Studying Fertility Behavior in Industrialized Populations

### Primary and Secondary Data Collection in Industrialized Populations

Our species exhibits so much behavioral flexibility, and lives in such a wide range of ecologies, that testing evolutionary hypotheses is always something of a challenge: truly powerful tests require data from the full range of human societies. Although human behavioral ecology, in particular, is built on an exceptionally strong foundation of detailed, in-depth studies of small-scale societies (see Winterhalder and Smith 2000 for a review), including information from industrial societies will give us greater power to identify variability in human fertility behavior, and the ecological variables that help generate this variation. Such societies have the advantage that many rich secondary datasets (i.e., data collected by someone else for a different primary purpose; Smith et al. 2011) are often available to test evolutionary hypotheses. Although there has been a long tradition of secondary data analysis using historical datasets in the human evolutionary sciences (Voland 2000), only recently has the discipline begun to exploit the large amount of existing data on contemporary industrialized populations (Nettle et al. 2013). We argue that such datasets are a valuable but underexploited resource in our discipline.

Evolutionary analyses of human behavior have traditionally focused largely on primary data collection, an approach that has many strengths. Most notably, primary data collection exercises can be designed to produce exactly the data needed to test a particular hypothesis, including both detailed surveys and experimental approaches (e.g., Henrich et al. 2005; Lamba and Mace 2011). Targeted studies of this kind also enable contextual, ethnographic, and qualitative detail to be gathered alongside quantitative data (e.g., Cooper 2014; Edin and Kefalas 2005). Primary data collection is therefore second to none in terms of providing a controlled and detailed view on the topic at hand.

Secondary data, in contrast, often suffer from the problem that it is not collected with a specific question of interest in mind, making it difficult to conduct an adequate and comprehensive test of a particular hypothesis. Despite this drawback, major positive arguments can be made in favor of secondary data (see Doolan and Froelicher 2009; Hofferth 2005; and Smith et al. 2011 for reviews on the use of secondary databases in distinct fields of research). First and foremost, secondary demographic, sociological, and/or epidemiological databases boast very large and often nationally representative samples and contain a wealth of information, typically including demographic, social, economic, and health information,^2^ though some focus in detail on specific topics (such as the UK’s National Survey of Sexual Attitudes and Lifestyles,^3^ and the UK’s Biobank,^4^ which has detailed health—including genetic—data). Many are also longitudinal, following the same individuals over time, allowing for in-depth investigations of life histories, including how early life experiences influence subsequent life events. Datasets also exist which allow comparative analysis, across both time and space. For example, there are now four UK birth cohort studies (with respondents born between 1946 and 2000), allowing comparisons between cohorts, and the Generations and Gender Survey is a comparative and longitudinal survey conducted across 17 European countries, Japan, and Australia, which allows between-country comparisons.^5^ In addition, since the process of data collection is “blind” to the hypotheses under study, it is less susceptible to confirmation bias.

In other words, although secondary data analysis is far from ideal (and certainly not the only means by which evolutionary researchers can study industrial economies), the wealth of large, representative secondary datasets does make it worthwhile for evolutionary researchers to investigate whether they can use this means to test their hypotheses before engaging in more expensive, time-consuming primary data collection (see Smith et al. 2011 for a similar view). This approach, of exploring secondary data first, may also help improve the power of primary data collection, as the latter can be targeted to fill the gaps in secondary datasets and produce the kinds of information that are not typically available in large-scale surveys (e.g., experimental work or detailed ethnographic data; see Daniel Nettle’s work on socioeconomic inequalities in the UK, which evolved from secondary data analysis [Nettle 2011] to fieldwork [Nettle 2015]).

Although the value of conducting studies on secondary data is clear, measuring and interpreting patterns of fertility behavior using secondary data is far from straightforward: the very complexity of such datasets means they also present a number of difficulties which may not be apparent if one lacks experience working with them. For an anthropologist facing the challenge of meticulously collecting primary data over months and years in the field, the analysis of secondary datasets, often downloadable at the click of a mouse from one of many online data repositories, may seem a trivial matter. Despite the ease with which secondary datasets can be acquired, it is important not to underestimate the amount of work needed to produce useful and comprehensible results. Here, we tackle several decisions that the individual researcher faces when dealing with a secondary database before going on to discuss some of the analytical problems that often arise but about which researchers can do little (except retain an awareness of the possibility for certain kinds of bias). Note that the issues we describe are inherent to all secondary data analysis but are particularly relevant to the analysis of the very large, complex datasets typical of industrialized populations.

### Challenges of Using Secondary Data: Researcher “Degrees of Freedom” and Population Heterogeneity

The nature of large, rich datasets, which contain an enormous array of information from a wide range of individuals, presents researchers with an equivalently large number of “degrees of freedom” (the choices made about which variables to analyze and how). This can allow inadvertent and unconscious bias to creep in. Before even embarking on any form of analyses, then, decisions must be made about (*i*) how to operationalize the research question—in other words, how to decide which variables will be used to measure the predictor and outcome; (*ii*) selection of the sample; and (*iii*) which pertinent (confounding) variables to include. The outcome of these decisions can (and often does) lead to different answers to the same research question. A case in point is a recent crowd-sourcing study that supplied an identical dataset and research question (Does football players’ skin color have an effect on the number of red cards issued to them by a referee?) to twenty-nine independent research teams and asked them to come up with an answer. This resulted in marked variability in both the analysis strategy and the statistical modeling approach used and, hence, to the answers produced (Silberzahn and Uhlmann 2015). Given that such wide variability is possible in a study with a limited set of variables and a single, straightforward research question, it is not surprising to find stark differences in research outcomes when a dataset contains thousands of variables, and when research questions are less well-defined. From a scientific viewpoint, then, it is vital that researchers fully explain the decisions they make during sample selection, and the variables they choose to include (or omit) (see statistician Andrew Gelman for a similar plea in psychology: Gelman and Loken (2014) and his blog).^6^ Even when researchers are fully aware of the possibility for bias and strive to avoid it, the formulation of particular constructs is often highly subjective.

Although large sample size is repeatedly emphasized as one of the major advantages of large demographic databases, sample sizes need to be large because of the heterogeneity that is common in industrialized populations. In studies of small-scale societies, it is reasonable to assume that the population is relatively homogenous, and to justify generalizations regarding reproductive strategies on this basis. In contrast, industrial settings are associated with various forms of social stratification, which potentially leads to heterogeneity in reproductive strategies. This applies both across populations (such that the United States may be markedly different from the United Kingdom), but also within populations (such as the distinction between rural and urban areas). A failure to account for such heterogeneity may lead to false inferences (Mace 2008; Pollet et al. 2015). For example, a recent study found that fewer children were born in wealthier urban areas of Mongolia than in poorer rural areas; yet, within both urban and rural areas, there was a positive association between resources and fertility (Alvergne and Lummaa 2014).

Heterogeneity in reproductive (or parental) strategies may exist on an even finer scale (e.g., across educational or socioeconomic strata; Kaplan 1996; Lawson and Mace 2011; Mace 2008; see also Stulp et al. 2016). Differences in partnership and reproductive strategies can be substantial in industrialized populations (not least because of the high degree of control afforded by modern contraceptive methods). For example, according to recent data from the UK, around half of women in the lowest wealth tercile were without a coresident partner at the time of their first birth, compared with almost none in the highest wealth tercile (Schaffnit 2015). Age at first birth also shows a clear, and increasing, educational gradient in the UK (Berrington et al. 2015): women born between 1960 and 1969 show a 7-year gap in median age at first birth for those in the highest and lowest educational groups, and a gap of about half a child in completed fertility between the two groups (highly educated women have later first births and a higher probability of childlessness). Although these patterns may, to some extent, simply reflect variation in reproductive patterns owing to variation across a particular dimension (e.g., access to resources), they may also reflect the existence of subgroups within populations whose reproductive decisions are influenced by rather different criteria (e.g., Borgerhoff Mulder 2007; Lawson and Mace 2009). These may include not just a difference in the salient environmental factors to which different subgroups are sensitive, but also differences in social influences and cultural norms surrounding reproduction (see Sweeney and Raley 2014 and Stulp et al. 2016 for the case of ethnic differences in partnership and reproduction).

The marked variability within industrial societies also suggests that samples may require more detailed description—more “ethnographic” detail—in order to uncover the variety of strategies likely to exist within a given population. This kind of detail is routine in evolutionary studies of small-scale societies, as well as the mixed methods and qualitative research typical of the social sciences (Bernardi et al. 2014; Cooper 2014; Edin and Kefalas 2005), and it is likely to substantially advance our (evolutionary) understanding of reproductive decision-making (see also Geronimus 1996). For example, ethnographic evidence suggests that poorer and less-educated families make their fertility decisions on the basis of short-term favorable circumstances, rather than on long-term (financial) prosperity (Cooper 2014; Edin and Kefalas 2005; see also Musick et al. 2009). In contrast, richer, more highly educated families, who are hypothesized to use their wealth as a buffer for future risk and uncertainty, postpone childbearing until they feel they have sufficient resources for having a (nother) child (Cooper 2014; Edin and Kefalas 2005; Musick et al. 2009).

### Family Influences on Fertility as an Illustrative Case

To illustrate the points made above, we offer the example of our work on whether family support is associated with fertility. Evolutionary researchers have shown interest in this topic because the cooperative breeding hypothesis (Hrdy 2009) makes the straightforward prediction that women who receive a lot of support from family should have more successful reproductive outcomes, including higher fertility. A number of studies have now investigated this issue in industrialized populations, but there is clear variability in the results, particularly within Europe: family support is observed to be both pro- and anti-natal, and sometimes support has no effect at all (see Schaffnit 2015 and Sear and Coall 2011 for reviews). Such variability could well be due to differences in institutional and cultural factors across these populations: industrialized populations are not a monolith. It is also likely, however, that such differences arise from the way that family support is measured in different datasets—support from both husband’s and wife’s family may be included, or different measures of support (survival status of family, coresidence with family, provision of practical support such as childcare, financial support, emotional support)—and how fertility is measured (age at first birth, length of birth intervals, total number of children or childlessness have all been investigated). The variability in Europe contrasts with studies conducted among industrialized populations in Asia, which show consistently that family support is associated with higher fertility. This may be because the Asian studies use similar variables to measure both family support and fertility: most investigate the association between coresidence with the husband’s family and length of interbirth intervals (reviewed in Schaffnit 2015).

Even within the same population, and with similar research questions, differences may be observed. For instance, Schaffnit (2015) and Mathews and Sear (2013) have investigated associations between family support and fertility in two UK datasets: the Millennium Cohort Study and the British Household Panel Survey, respectively. In the latter, childcare from family members was associated with a faster progression to second births; in the former, there was much weaker evidence that grandparental childcare was correlated with a higher probability of second birth. One possible explanation for these differences may be differences in the way that childcare data were collected. In the BHPS, women were asked to report who looked after their children “while you are at work,” meaning such data was only reported by women employed outside the home; the variable included in the analysis also incorporated childcare from any relative, which was likely, but not necessarily, to be care provided by grandparents. In contrast, the MCS contains data from all women on the receipt of childcare from both sets of grandparents, collected soon after birth. Without a close inspection of how data on family-provided childcare was collected in each dataset, it would appear that an ostensibly identical research question (is family-provided childcare associated with higher fertility in the UK?) inexplicably leads to different results in the two studies.

This same MCS study also found that different types of support have contrasting effects on fertility: while emotional support was associated with a *higher* probability of second birth, receiving financial support was associated with a *lower* probability of second birth (perhaps because the receipt of financial support from kin is associated with greater need on the part of recipients, rather than being an indication that these individuals have plentiful support they can convert into childbearing; e.g., Schaffnit and Sear 2014). Different measures of support are clearly not interchangeable when the influence of family on fertility is being investigated. Such results suggest that singling out particular variables and neglecting others may provide a biased and somewhat misleading picture of the patterns at hand. Another recent study using the MCS, for instance, showed that contact frequency with parents-in-law predicted faster progression to second birth (Tanskanen et al. 2014)—results that were interpreted as potentially consistent with the cooperative breeding hypothesis. Although they provide detailed description of their methods and the analyses themselves are exemplary, carefully distinguishing the influence of parents and parents-in-law and separating the analysis by parity, it is not immediately apparent why contact frequency was chosen as the *sole* measure of family support, and why measures of the provision of actual support by kin were excluded. Reporting why certain measures are used and not others, and justifying these choices, would be a valuable addition to such analyses, allowing researchers to more fully understand why variable patterns emerge from equivalent datasets.

Finally, research on family and fertility also highlights how within-population heterogeneity should be taken into consideration when analyzing secondary datasets. Although the MCS analysis showed little evidence for an association between grandparental childcare and higher fertility overall, when the dataset was stratified by socioeconomic status there was evidence for a positive relationship between such childcare and fertility in the lowest socioeconomic tercile, but not in the middle or upper tercile.

### Data Quality and Biases

In addition to the challenges that arise from “researcher degrees of freedom” and population heterogeneity, there are also issues of data quality and bias (for recent examples, see Kreyenfeld et al. 2013; Sauer et al. 2016). Such biases are certainly not restricted to secondary databases; they hold generally for any study with human subjects. However, when a researcher has not been involved in the collection of a particular dataset, the flaws may not be obvious in the way that they are when data are collected by researchers themselves. It is easy to assume, for example, that data have been collected both consistently and comprehensively across time in longitudinal studies. In reality, questions can be reworded to produce slightly different answers or targeted at a slightly different subset of respondents. Moreover, questions can be dropped from surveys altogether, or new ones added. Additionally, not all the variables needed to test a given hypothesis or control for confounds may be collected.

Concerns about bias and data quality are, of course, amplified for those interested in performing comparative analyses across several secondary datasets at once since information on the area of interest may have been collected using different questions in different populations. Some multi-country datasets, however, are explicitly designed to allow comparative analyses, such as the Gender and Generations Programme mentioned above. Although the surveys used in each country are designed to be as similar as possible to allow comparative research, there are still differences between countries in how the surveys were conducted, the exact questions asked, and how questions were translated and/or interpreted in different countries. Nevertheless, such cross-national datasets can be useful in comparative research, provided sufficient care is taken over the analysis and interpretation of results.

Another worry is that of response bias and selective sampling and, in cases of longitudinal follow-up, the attrition of respondents through time. Although demographic surveys aim to be nationally representative, some people, such as those with low income, tend to be harder to capture and more likely to drop out of surveys (e.g., Goyder et al. 2002; Groves 2006; Strandhagen et al. 2010). Such biases may have serious implications for fertility research, in particular, and for the conclusions that can be drawn in an evolutionary framework. For example, studies have shown that men under-report pregnancies from previous relationships, and that previously married and unmarried men are less likely to be included in demographic samples, leading to sex differences in the number of reported children (Rendall et al. 1999; Stulp et al. 2012a; see Stulp et al. 2016 for more examples). Another potential bias may arise through extra-pair paternity: men may not know they are the father of certain children, whereas other men may erroneously assign paternity to children who are not, in fact, their own. Although this problem needs to be acknowledged, it seems unlikely that such errors will substantially affect outcomes because extra-pair paternity rates are very low (Anderson 2006).

In a different vein, when sampling at older ages, biases resulting from death or non-random dropout should be taken into account: when collecting a sample of older individuals only, or when asking about certain variables only at older ages, there will be a bias toward people who have survived and thus remain in a position to respond to the question. Such (potential) biases should not be neglected, as they pose a real problem for studies of reproductive decision-making. Research on height provides an informative case. Height is associated with reproductive success in many populations, but it is also associated with mortality, the likelihood of getting married, and income (see Sear 2010; Stulp and Barrett 2016a; Stulp et al. 2012b, c for reviews), all of which may bias the estimates we observe for the association between height and reproductive success. For instance, in the Wisconsin longitudinal study, height was only recorded once the participants had reached the age of 52 (Stulp et al. 2012a, b, c). Given that unmarried and deceased people are, respectively, less likely and certain not to be included in follow-up studies, and that both of these factors are also associated with height, our sample at later ages may be biased with respect to this trait. Moreover, the respondents in this study had all graduated from high school, which, given the positive association between height and education, potentially biases the sample toward taller heights (Stulp et al. 2012b). Similarly, in another study, we found a high incidence of individuals unwilling to report on their income (Stulp et al. 2015). Again, given the association between height and income, it is likely that our sample may be somewhat biased in this respect.

Another concern relates to the accuracy of the data collected. Large-scale surveys typically make substantial demands on respondents because of the large number of questions, and the sensitivity of some of those questions. Some respondents may simply refuse to answer anything that makes them uncomfortable, which leads to a straightforward response bias (e.g., with respect to wealth questions: Ross and Reynolds 1996; Zagorsky 1999). A more difficult issue is raised by respondents who answer these sensitive questions, but do so inaccurately. One means of reducing such biases is to incorporate data collection techniques such as self-completion questionnaires (which allow respondents to answer questions via computer screen, rather than respond directly to an interviewer). Problems of accuracy still persist, however, in cases where people simply do not know the correct answer to a given question (whether sensitive or not). There is a marked tendency for people to underestimate their wealth (Zagorsky 2000), for example, and they are also not particularly confident about their partner’s income (see Stulp et al. 2016).

Finally, some problems inherent to large questionnaire surveys are almost impossible to avoid or ameliorate. Survey methodologists are aware, for example, that “context effects” can cause problems: preceding questions may influence the interpretation of, or responses to, subsequent questions (Todorov 2000; Tourangeau et al. 2000, 2003). This may have relevance for fertility research, particularly if researchers are interested in analyzing data on fertility preferences, which are widely collected in surveys but have been shown to be influenced by priming effects (Mathews and Sear 2008; Wisman and Goldenberg 2005). In the first wave of the UK Millennium Cohort Survey, for example, women were asked questions about their future fertility intentions immediately after questions about their previous birth, including whether pain relief had been used. Such ordering is potentially problematic; priming women to think about the pain of childbirth may lead to (temporarily) reduced future fertility intentions (Mathews 2012). Experimental studies show that the ordering of questions matters in fertility research (Mathews 2013).

None of the above should be taken to mean that secondary data analyses raise any more problems that those encountered when analyzing data that researchers have collected themselves. Problems of response bias, selective sampling, and question-ordering effects are likely in all datasets. The point is that such problems may be less obvious if researchers have not encountered them at first-hand during data collection, and efforts should be made to be aware of these issues and consider their potential impacts on the design and outcome of our analyses.

## Conclusion

By highlighting the value of studying fertility in industrial populations, while also recognizing the challenges of doing so, we have aimed to provide evolutionary anthropologists with a well-informed incentive to embark on a more comprehensive examination of the reproductive ecology of industrial societies. Although differences in data collection between datasets may complicate the interpretation of relationships of interest, they also open up the possibility of testing alternative hypotheses, allowing us to gain a more sophisticated understanding of fertility behavior and how it might vary across groups.

We hope, therefore, to have persuaded the reader that measuring fertility does matter, that there is such a thing as the reproductive ecology of industrial societies, and that industrial populations, in all their variety, need to be incorporated into an evolutionary framework. Industrial populations should be seen as a necessary addition to a fully comprehensive understanding of human (mal) adaptive behavior, and not as an optional extra.

## Footnotes

1 Although our use of the term “fertility” conforms to the definition used in the social sciences (i.e., the number of children born) rather than the biological definition, which considers fertility to be the ability to conceive, we extend beyond this strict definition to encompass all aspects of reproductive behavior in our discussion of why measuring fertility is important.

2 See https://www.data.gov/ for open access datasets from the US; http://www.data-archive.ac.uk/ for UK datasets; https://www.icpsr.umich.edu/ for an overview of a large number of datasets available worldwide

3
http://www.natsal.ac.uk/home.aspx

4
http://www.ukbiobank.ac.uk/

5
http://www.ggp-i.org/

6
http://andrewgelman.com/
